# Supplementary material for: Deciphering the factors influencing electric field mediated polymerization and depolymerization at the solution–solid interface
Source: Commun Chem. 2024 May 9;7:106. doi: 10.1038/s42004-024-01187-2 (PMC11082217; doi:10.1038/s42004-024-01187-2)
Supplement: Supplementary file 2 — Supplementary information [file 42004_2024_1187_MOESM2_ESM.pdf]

Supplementary information

Deciphering the Factors Influencing Electric Field Mediated Polymerization and Depolymerization at the Solution-Solid Interface

Zhinan Fu, Nicolás Arisnabarreta, Kunal S. Mali, and Steven De Feyter**

Division of Molecular Imaging and Photonics, Department of Chemistry, Celestijnenlaan 200F, Leuven, Belgium-3001

Email: kunal.mali@kuleuven.be; steven.defeyter@kuleuven.be

CONTENTS:

1. Initial STM scans at positive substrate bias showing 2DP domains at the HA/graphite and OA/graphite interface **(Figure S1)**.
2. STM images showing the bias-induced (de)polymerization of TPBA at the OA/graphite interface **(Figure S2)**.
3. Time-dependence of depolymerization within the scanned area at the MO/graphite interface **(Figure S3).**
4. Representative STM image showing the co-existence of 2DP and SAMN at the TCB/graphite interface at negative sample bias **(Figure S4)**.
5. Representative STM images showing the formation of SAMN of TPBA at the PO/graphite interface positive as well as negative substrate bias **(Figure S5)**.
6. Impact of added water on the observation of 2D polymer at the HA/graphite interface **(Figure S6)**.
7. Additional data obtained upon continued scanning at positive sample bias in continuation of STM scans provided in Figure 3 in the main text **(Figure S7)**.
8. Time-dependence of depolymerization within the scanned area at the OA/graphite interface **(Figure S8).**
9. STM images showing the self-assembly of covalent trimers (**SAMN1**) observed at the HA/graphite interface **(Figure S9)**.
10. Additional STM images showing desorption of monomers at negative substrate bias at the HA/graphite interface **(Figure S10)**.
11. Additional STM data showing negative bias-induced desorption and positive sample bias-induced adsorption of TPBA molecules at the OA/graphite interface **(Figure S11)**.
12. Additional STM data showing the depolymerization of 2DP at positive substrate bias as a function of time at the OA/graphite interface **(Figure S12).**
13. Representative STM images showing the effect of continuous negative scanning at negative bias **(Figure S13)**.
14. Additional and supporting STM data showing the dynamic processes occurring at the solution-solid interface during STM scanning **(Figure S14, S15, S16).**

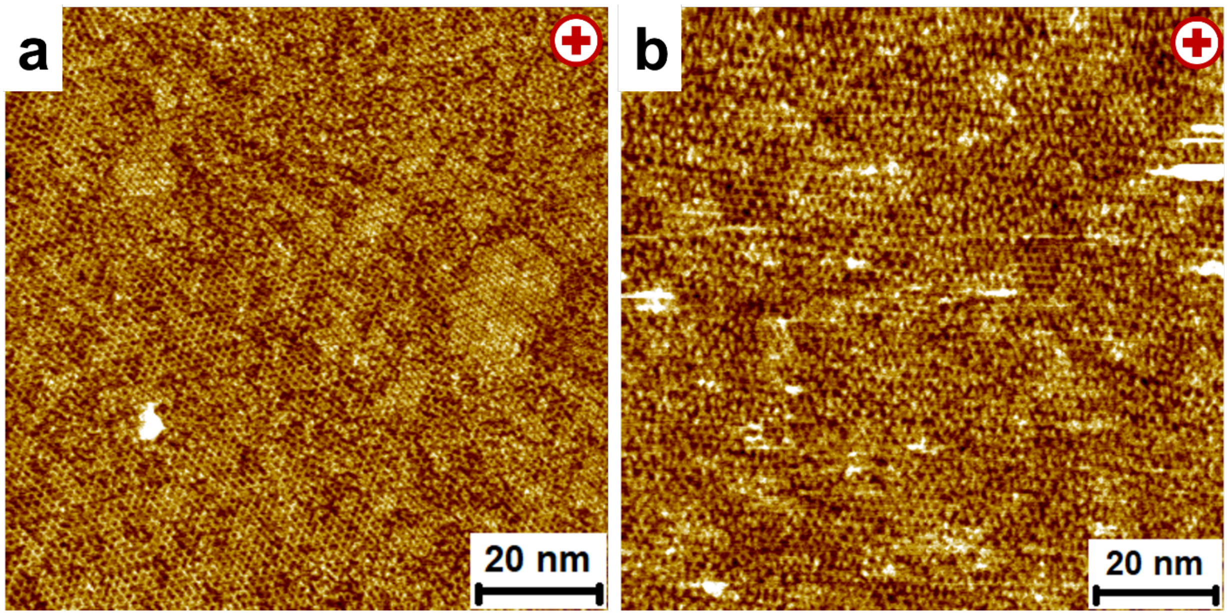

Figure S1. Representative STM images showing the TPBA networks formed at the (a) HA–graphite, and (b) OA–graphite interface when the surface was scanned at positive sample bias. Domains of the 2DP can be readily observed in these initial scans. Imaging conditions: I_{set} =0.1nA, V_{bias} =+0.7 V. [TPBA]=250μM.

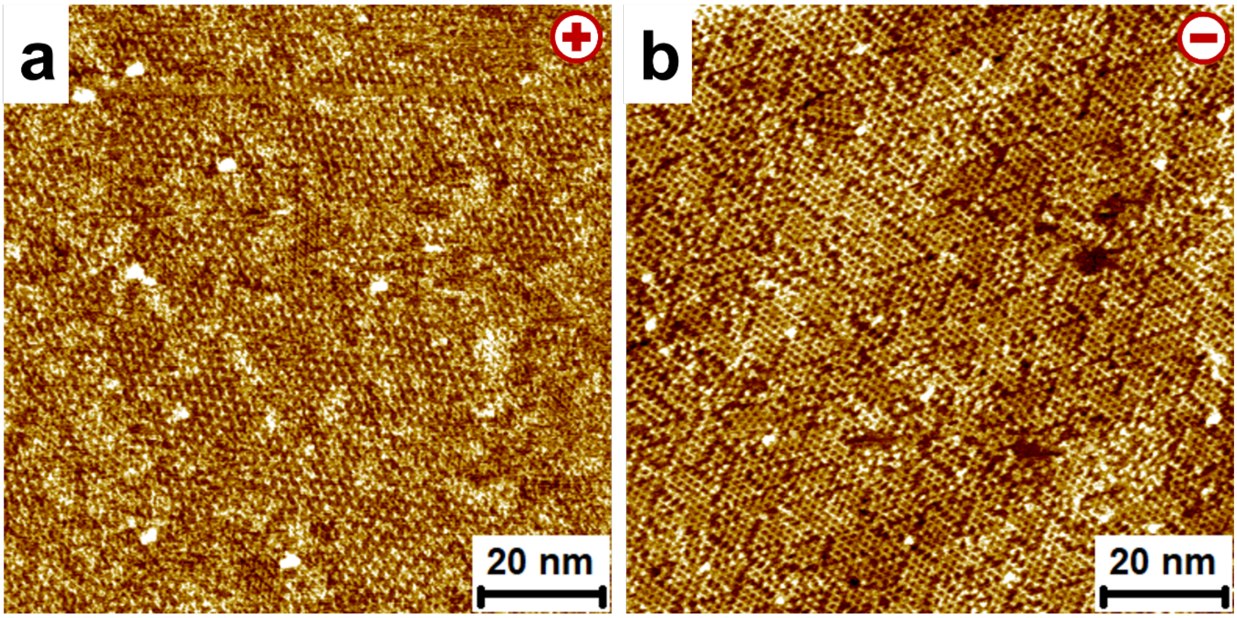

Figure S2. Representative STM images showing bias-induced (de)polymerization of TPBA at the OA–graphite interface. (a) SAMN formed at positive sample bias. Imaging conditions: I_{set} = 0.1 nA, V_{bias} = + 0.7V. (b) Boroxine-linked 2DP observed at the negative substrate bias. Imaging conditions: I_{set} = 0.1 nA, V_{bias} = – 0.7V. [TPBA] = 50 μM.

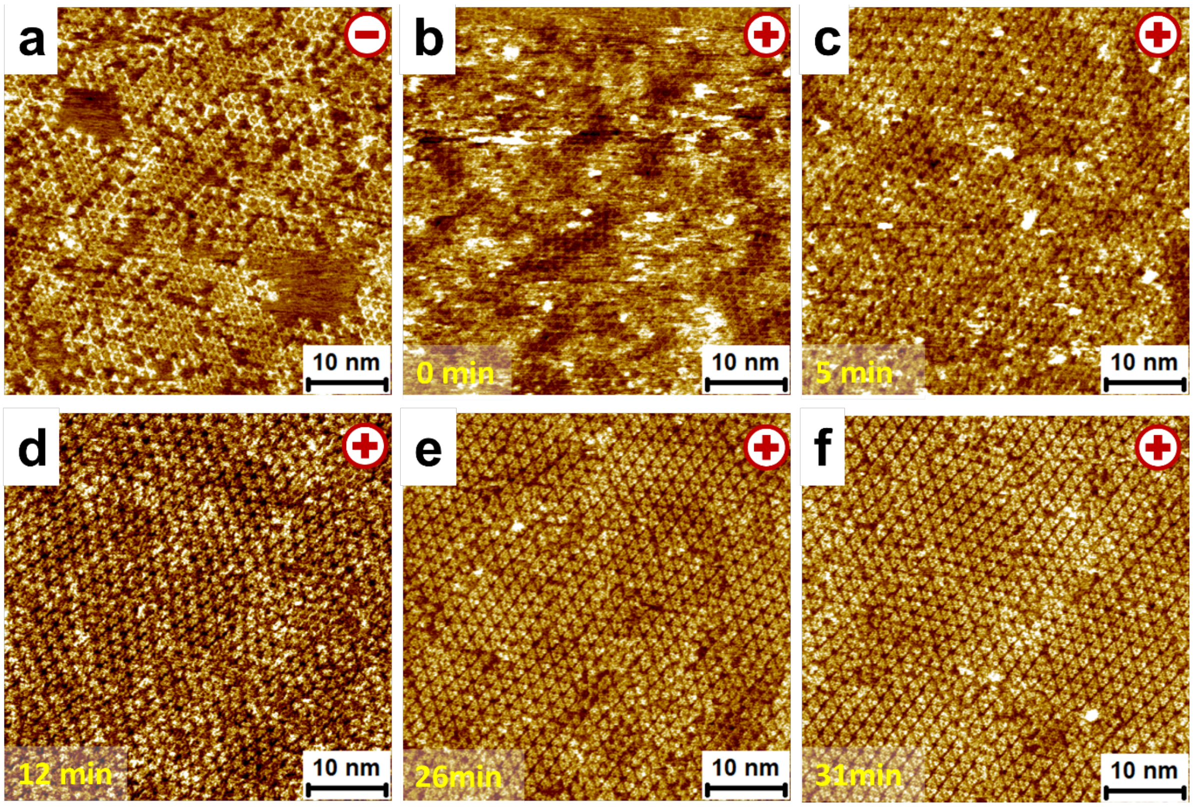

Figure S3. Time-dependence of depolymerization within the scanned area at the MO–graphite interface at [TPBA] = 250 μM. (a) STM image showing 2DP at negative sample bias within the scanned area (50 × 50 nm^2). (b) STM image showing the monolayer structure of TPBA immediately after switching the sample bias from negative to positive. (c-f) Sequential STM images showing the depolymerization process (50 × 50 nm^2) by continuous scanning at positive sample bias. Imaging conditions: I_{set}=0.1nA, V_{bias}=– 0.7V or + 0.7V.

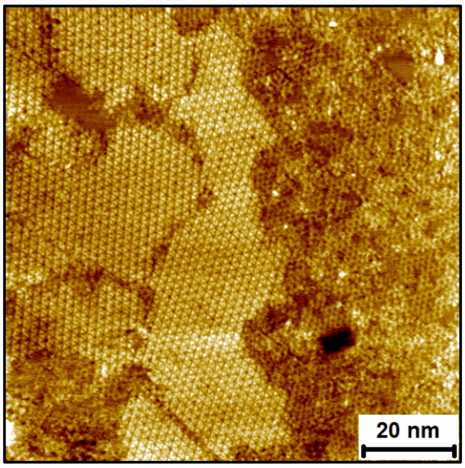

Figure S4. Representative STM image showing the co-existence of 2DP and SAMN at the TCB–graphite interface at negative sample bias. We hypothesize that the SAMN is formed on top of the 2DP which is adsorbed on the graphite surface. Imaging conditions: I_{set} = 0.1 nA, V_{bias} = – 0.7V. [TPBA] = 250 μM.

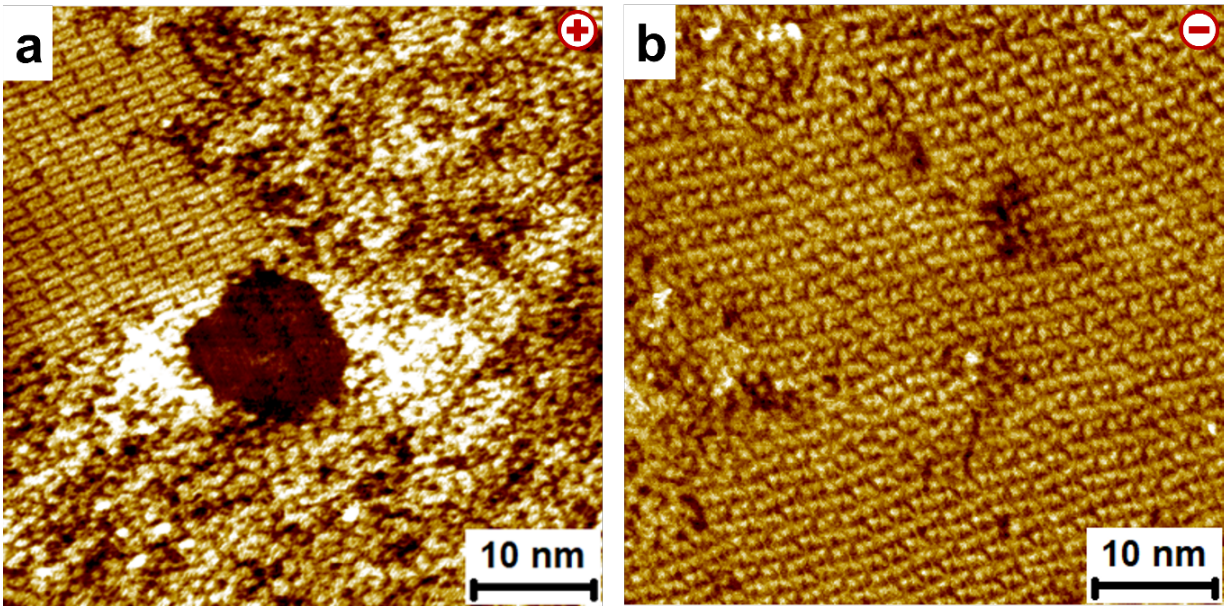

Figure S5. Representative STM images showing the SAMN of TPBA at the PO–graphite interface at (a) positive sample bias, and (b) negative sample bias. Imaging conditions: I_{set} =0.1nA, V_{bias} = – 0.7V or + 0.7V. [TPBA]=250μM.

To investigate the impact of water present in the system, we also conducted experiments involving the controlled addition of water to the solvent. Initially, a 50 μM TPBA-HA solution was prepared following the previously established method. Subsequently, this solution was mixed thoroughly with deionized water at volumes of 3%, 7%, and 10%, respectively. The results of the STM experiments are presented below.

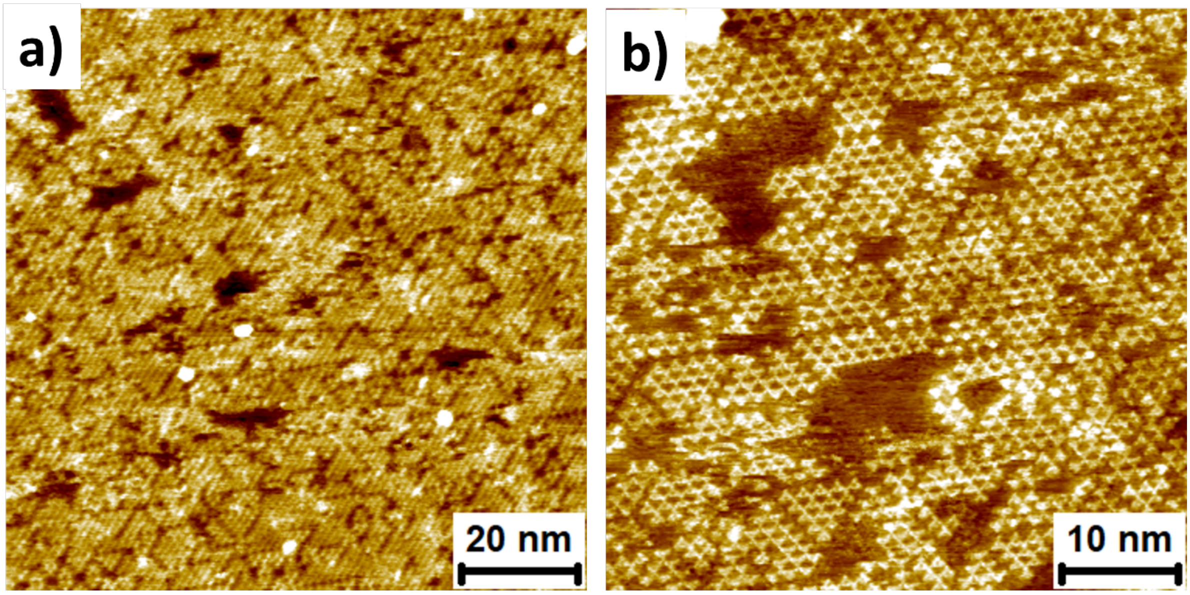

Figure S6. Representative STM images showing the 2DP at the HA+7%H_2O–graphite interface at negative sample bias. Imaging conditions: I_{set} =0.1nA, V_{bias} = – 0.7V. [TPBA] = 50μM. At positive substrate bias, however, we could not obtain molecular resolution.

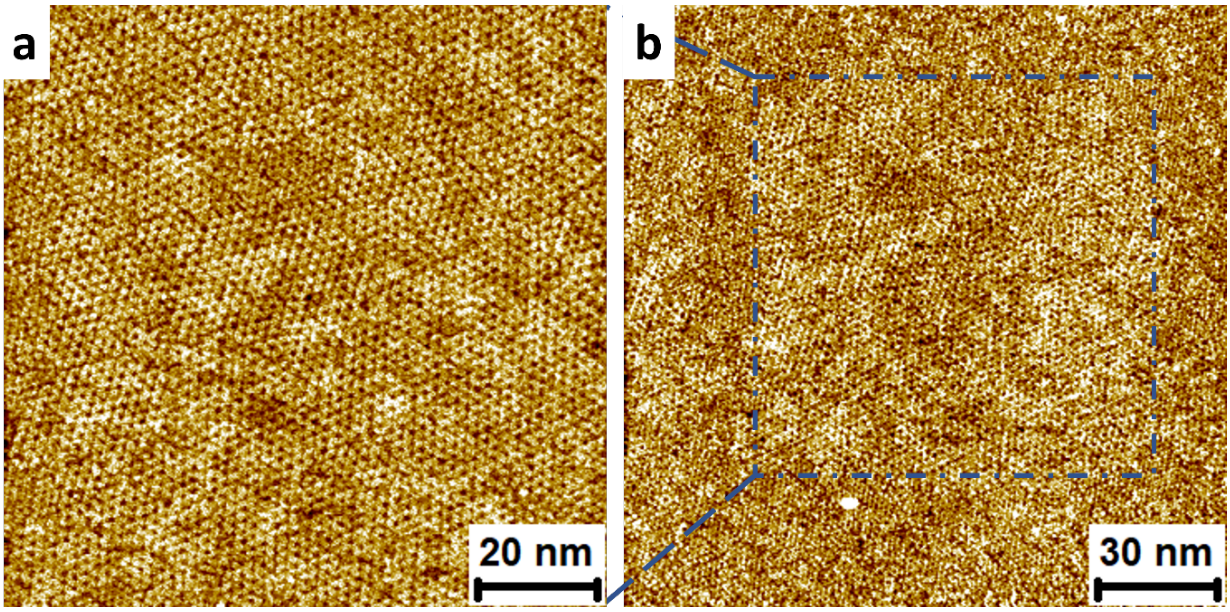

Figure S7. (a) A large-scale image (100 × 100 nm^2) obtained after continuous scanning at positive sample bias in the same general area as Figure 4(h) in the main text. (b) A larger scale image (150 × 150 nm^2) obtained after zooming out from (a) shows the local depolymerized region (blue square). The region outside of the scanned area still shows the presence of 2DP. Imaging conditions: I_{set} = 0.1 nA, V_{bias} = – 0.7V or + 0.7V. [TPBA] = 250 μM.

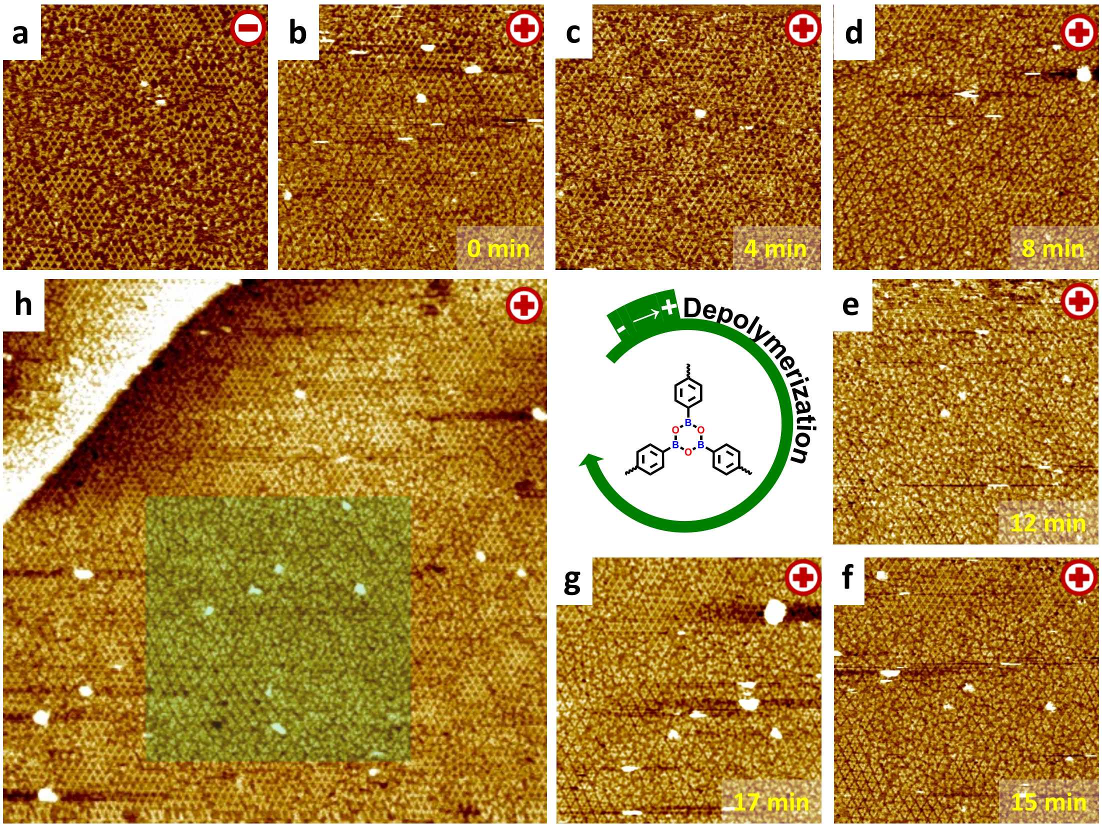

Figure S8. Time-dependence of depolymerization within the scanned area at the OA–graphite interface at [TPBA] = 250 μM. (a) STM image showing 2DP at negative sample bias within the scanned area (50 × 50 nm^2). (b) STM image showing the monolayer structure of TPBA immediately after switching the sample bias from negative to positive. (c-g) Sequential STM images obtained in the same general area as (a, b) showing the local depolymerization process (50 × 50 nm^2). (h) A larger scale image obtained after zooming out from (g) shows the locally depolymerized region (green square). The region outside of the scanned area still shows the presence of 2DP (100 × 100 nm^2). Imaging conditions: I_{set} = 0.1 nA, V_{bias} = – 0.7V or + 0.7V.

Session-to-session variations:

Some session-to-session variations in the time scale of the polymerization and depolymerization process were observed. For example, the time required for bias-dependent polymerization while scanning an area of 50 nm × 50 nm differed by up to 50% in two different sessions. This was observed for both octanoic as well as heptanoic acid. Similar differences were observed for the depolymerization process as well. These differences could be possibly related to the size of the domains of the SAMN (or the 2DP) at the point of bias-switch and the overall stability of the STM feedback. One expects a larger domain to be more stable and thus take longer to depolymerize/desorb at the liquid-solid interface. We emphasize that while there is variation in the time scales from session to session, in general, the depolymerization process was always found to be slower than the 2D polymerization process.

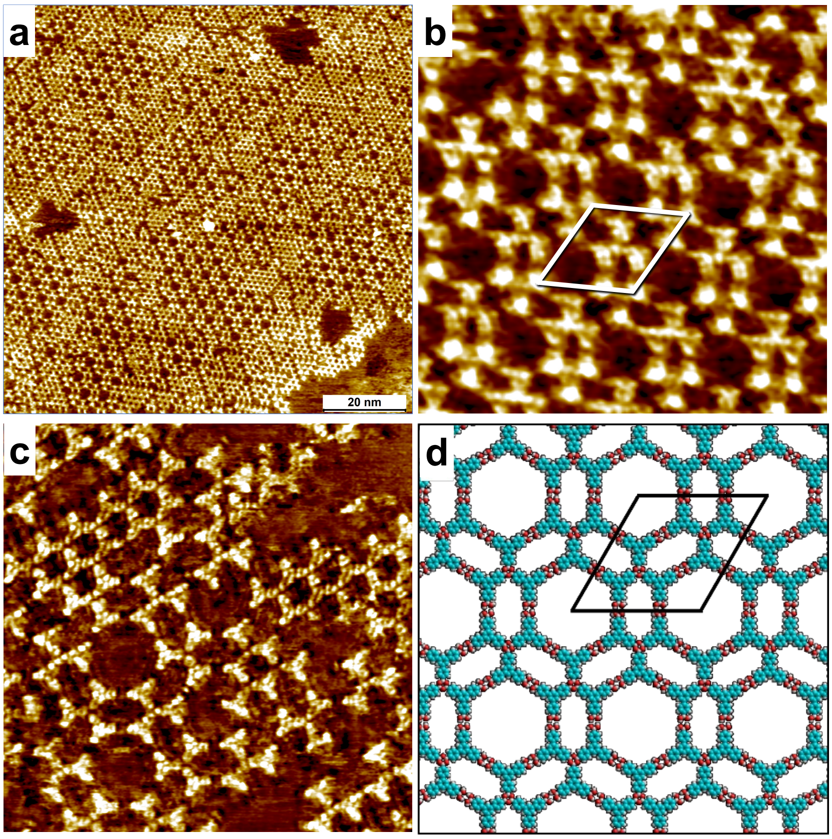

Figure S9. (a-c) STM images showing the self-assembly of covalent trimers (**SAMN1**) observed at the HA–graphite interface. Scan size (b) 19 × 19 nm² and (c) 20 × 20 nm². (d) A molecular model depicting the packing arrangement within SAMN1. Imaging conditions: I_{set} = 0.1 nA, V_{bias} = – 0.7V. [TPBA] = 25 μM.

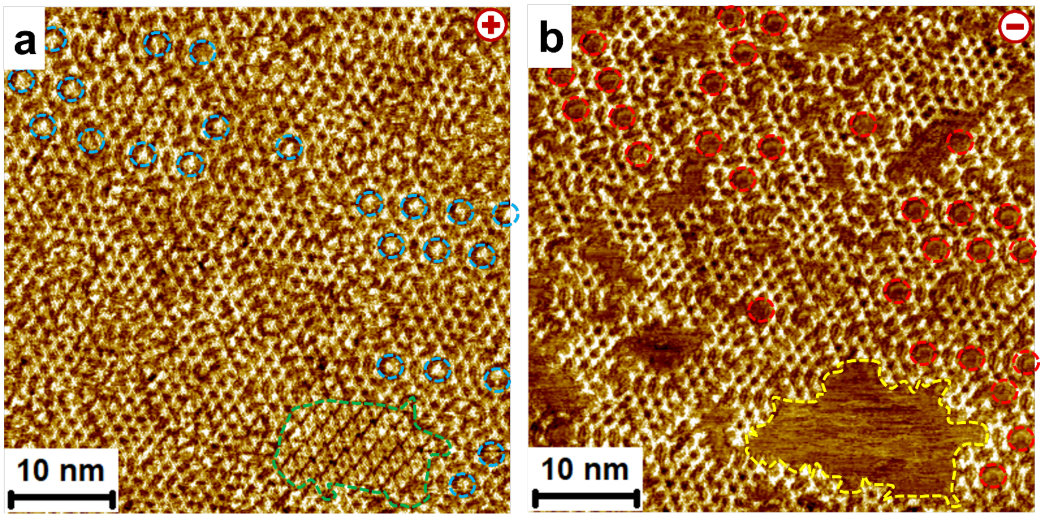

Figure S10. STM images showing desorption of monomers at negative substrate bias at the HA–graphite interface. (a) STM image at positive substrate bias where single TPBA molecules are adsorbed in the pore of trimer assembly. (b) Image obtained immediately after switching the sample bias from negative to positive, showing the desorption of non-covalently bonded molecules. Imaging conditions: I_{set} =0.1nA, V_{bias} = – 0.7V or + 0.7V. [TPBA]= 25μM.

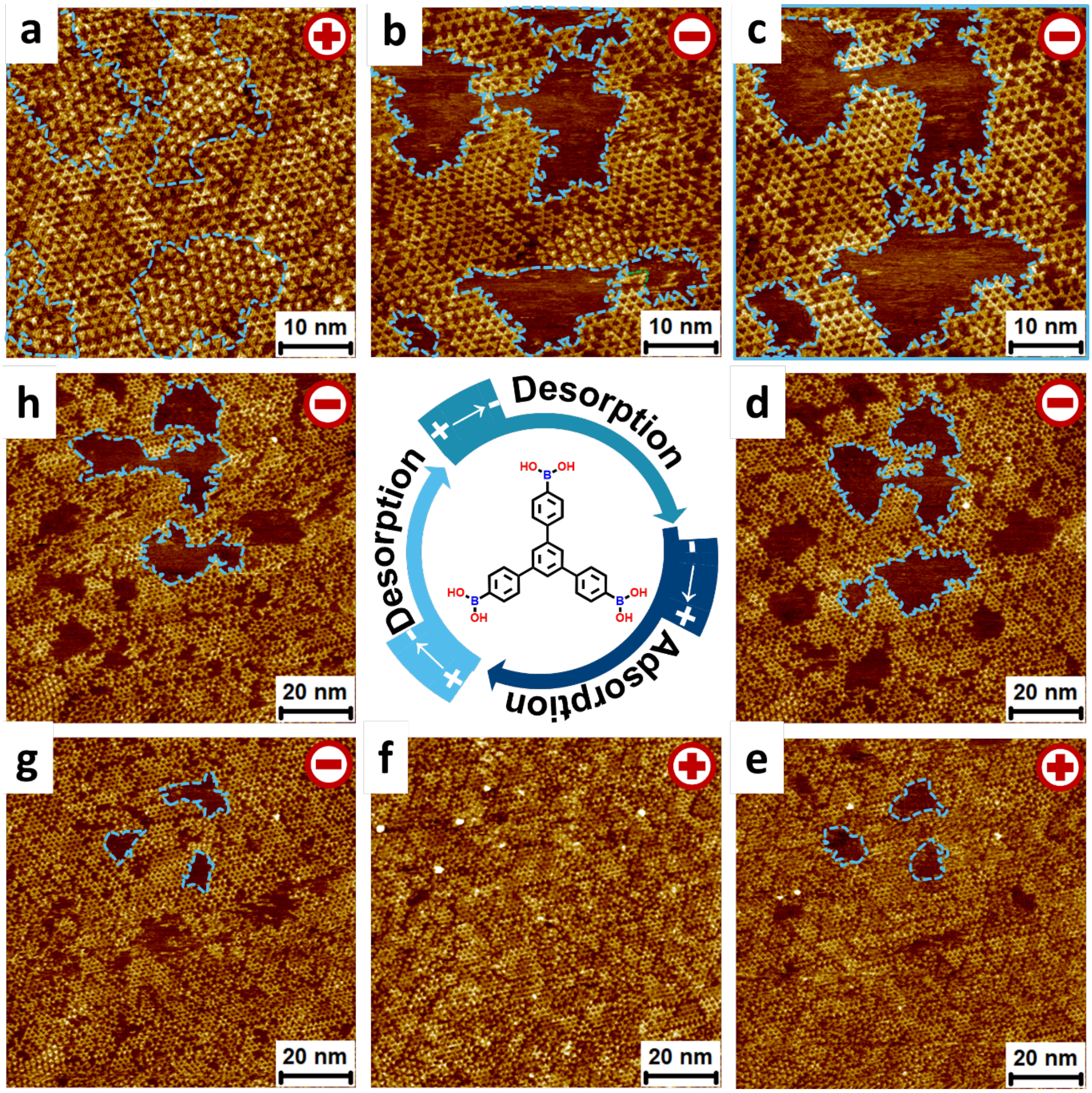

Figure S11. Representative STM images showing negative bias-induced desorption and positive sample bias-induced adsorption of TPBA molecules at the OA–graphite interface. (a) the mixture of 2DP and SAMN at positive bias; (b-d) sequential STM images showing the desorption of unpolymerized molecules induced by changing sample bias to negative; (e, f) gradual adsorption of molecules induced by changing sample bias to positive; (g, h)sequential STM images showing the desorption of TPBA molecules induced by changing sample bias to negative. Imaging conditions: I_{set} =0.1nA, V_{bias} = – 0.7V or + 0.7V. [TPBA]= 50μM.

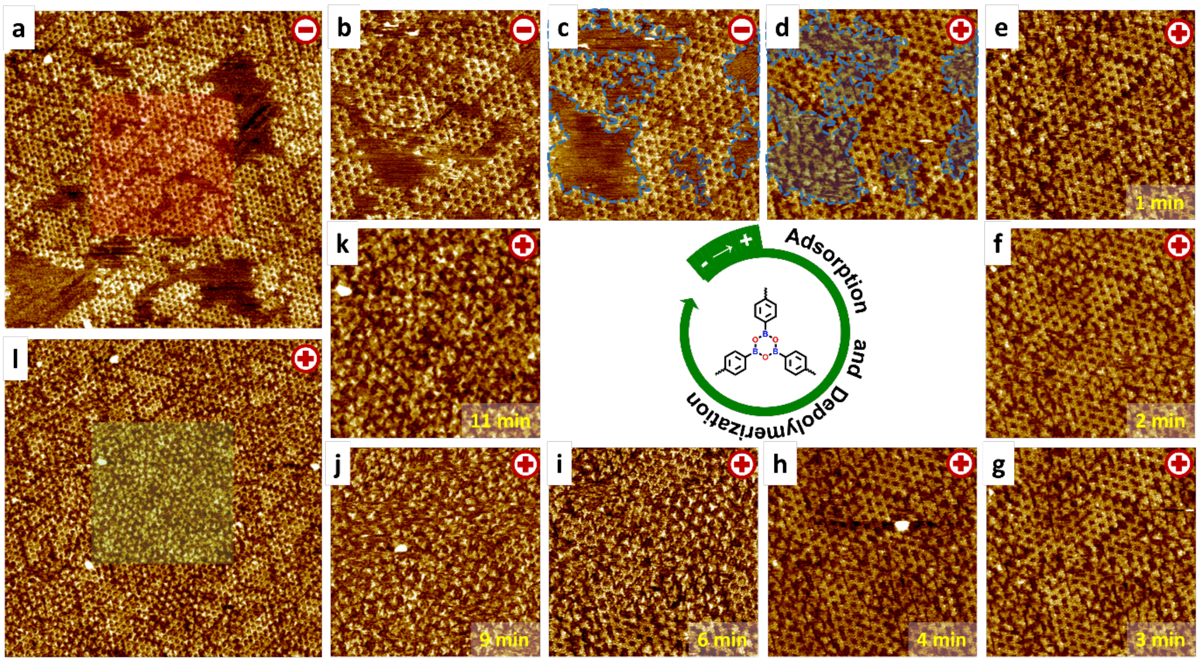

Figure S12. STM images at OA–graphite interface showing: (a) 2DP at a large-scale area at negative sample bias(75 × 75 nm^2); (b, c) zoomed-in images(30 × 30 nm^2) from the red region in (a) showing 2DP at negative sample bias; (c-k) sequential adsorption and depolymerization processed induced by changing sample bias to positive; (l) Large-scale STM image (75 × 75 nm^2) captured immediately after recording the small area STM images shown in (a-k), demonstrating local depolymerization within the long-term scanned region (green rectangle), while the surrounding region (outside the green rectangle) retains the 2DP. Imaging conditions: I_{set} =0.1nA, V_{bias} = – 0.7V or + 0.7V. [TPBA]= 50μM.

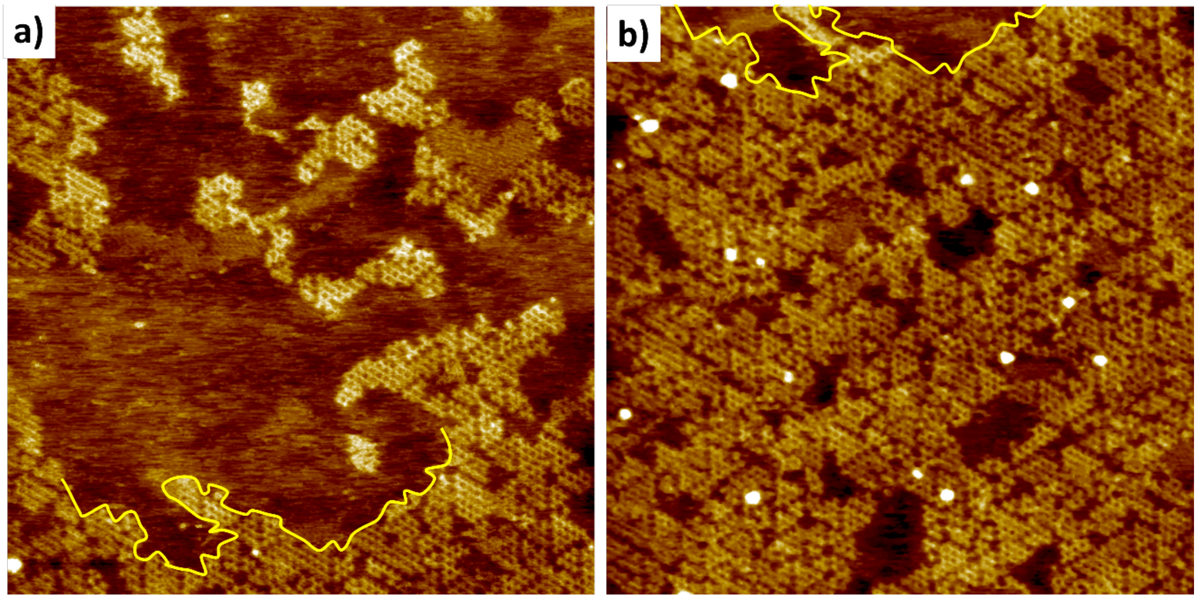

Figure S13. Representative STM images showing the effect of continuous negative scanning at negative bias. (a) First scan after switching the bias from positive to negative bias. (b) Last scan after continuous scanning at negative bias. The images in between these two scans are presented as time-lapse video as video (Supplementary video 1). The video shows dynamic transitions occurring within the surface adsorbed structures (continuous scanning for 81 minutes) including the making and breaking of covalent bonds as well as adsorption-desorption dynamics. Imaging conditions: I_{set} =0.1nA, V_{bias} = – 0.7V. [TPBA] = 50μM, Image size = 100×100 nm^2

Additional STM depicting the dynamic processes occurring at the solution-solid interface in response to continuous scanning at a given sample bias.

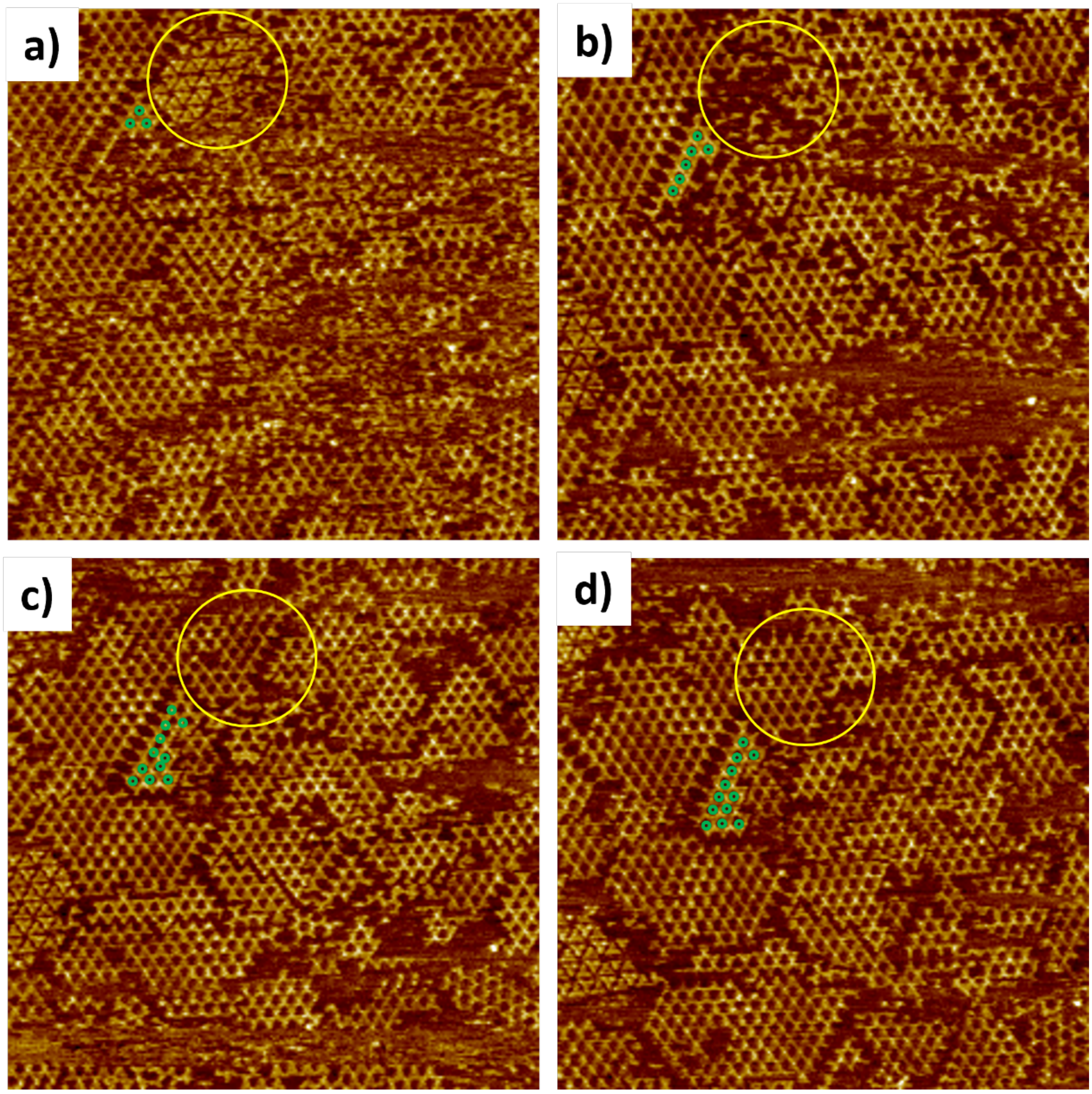

Figure S14. Sequential STM images (50 × 50 nm^2) obtained of TPBA monomers at the HA–graphite interface show the dynamic process, including adsorption, desorption, and polymerization at negative sample bias. Imaging conditions: I_{set} =0.1nA, V_{bias} = – 0.7V. [TPBA]= 250μM.

At negative sample bias, molecules also adsorb onto the surface and given sufficient space, the molecules can rotate. As illustrated in the triangular region in panel a, the arrow indicates a missing molecule, and in b, a molecule has adsorbed. However, due to the different orientation of the newly adsorbed molecule compared to the pre-existing 2DP, the attachment is not immediate. In panels (c) and (d), it can be seen that the newly adsorbed molecule adjusts its orientation and integrate with the small 2DP domain.

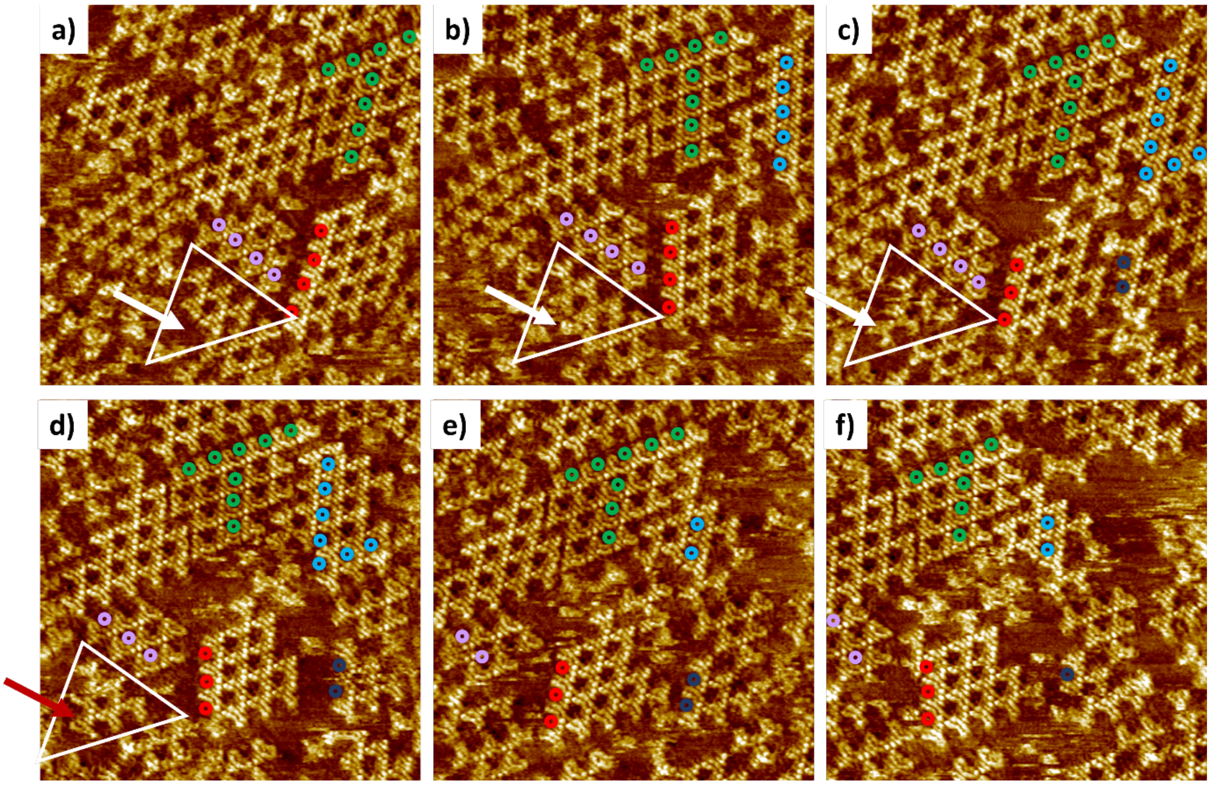

Figure S15. Representative STM images showing polymerization dynamics of TPBA at the OA–graphite interface at negative bias in consecutive images. Imaging conditions: I_{set} =0.1nA, V_{bias} = − 0.7V. [TPBA] = 50 µM, Image size = 50 × 50 nm^2

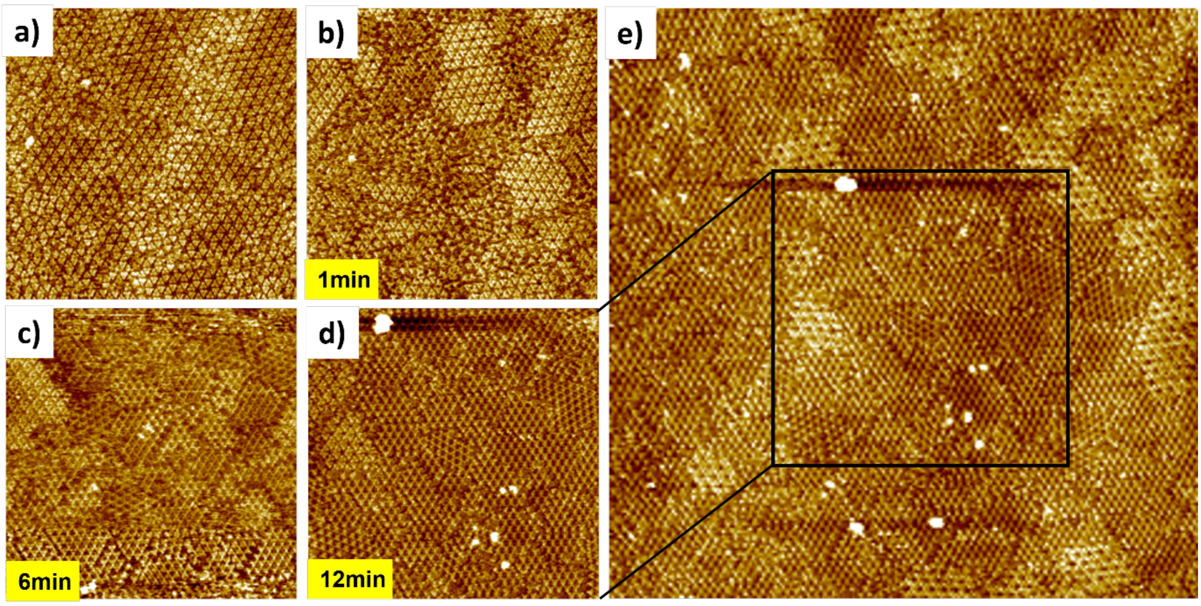

Figure S16. Time-dependence of polymerization within the scanned area at the HA–graphite interface at [TPBA] = 250 μM. Starting with SAMN observed at positive sample bias in (a), this image sequence shows how the domains of SAMN are removed upon scanning at negative bias (b-d) with subsequent formation of 2DP (50×50 nm^2). (e) A larger scale image obtained after zooming out from (d) shows polymerization doesn't happen locally. The region outside of the scanned area still shows the presence of 2DP (100×100 nm^2). Imaging conditions: I_{set} =0.1nA, V_{bias} = – 0.7V or + 0.7V. [TPBA]= 250μM.

Orientation of the unit cells with respect to symmetry axes of graphite lattice

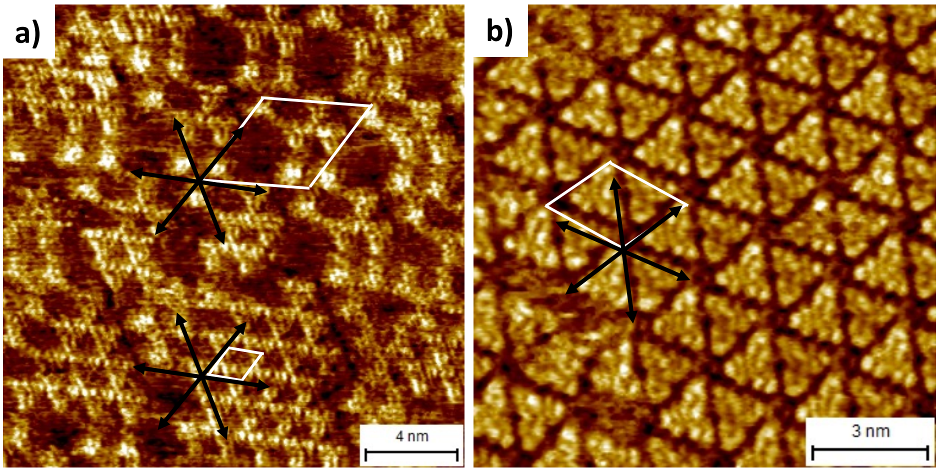

Figure S17. STM images calibrated using the graphite lattice showing the orientation of 2DP, SAMN1 (a), and SAMN (b) with respect to the symmetry axes of graphite. The unit cell vectors of the 2DP domains were found to be coincident with the symmetry axes of graphite lattice. The unit cell of the SAMN1 network was oriented at a small (~3°) angle with respect to the symmetry axes of the underlying graphite lattice. Lastly, the SAMN unit cell was rotated by ~8° with respect to one of the symmetry axes of the graphite lattice.
