# Supplementary material for: Deciphering the factors influencing electric field mediated polymerization and depolymerization at the solution–solid interface
Source: Commun Chem. 2024 May 9;7:106. doi: 10.1038/s42004-024-01187-2 (PMC11082217; doi:10.1038/s42004-024-01187-2)
Supplement: Supplementary file 3 — Description of Additional Supplementary Files [file 42004_2024_1187_MOESM3_ESM.pdf]

Description of Additional Supplementary Files

File name: Supplementary Video 1

Description: A video showing the effect of continuous scanning at negavtive polarity of the sample bias.
